# Oral Candidiasis among Cancer Patients Attending a Tertiary Care Hospital in Chennai, South India: An Evaluation of Clinicomycological Association and Antifungal Susceptibility Pattern

**DOI:** 10.1155/2016/8758461

**Published:** 2016-06-14

**Authors:** Abirami Lakshmy Jayachandran, Radhika Katragadda, Ravinder Thyagarajan, Leela Vajravelu, Suganthi Manikesi, Shanmugam Kaliappan, Balaji Jayachandran

**Affiliations:** ^1^Department of Microbiology, Karpaga Vinayaga Institute of Medical Sciences and Research Center, Madurantakam Taluk, Kanchipuram, Tamil Nadu 603308, India; ^2^Department of Microbiology, Government Kilpauk Medical College Hospital, Chennai, Tamil Nadu 600010, India; ^3^Department of Microbiology, King Institute of Preventive Medicine, Guindy, Chennai, Tamil Nadu 600032, India; ^4^Department of Prosthodontics, Indira Gandhi Institute of Dental Sciences, Puducherry 607402, India

## Abstract

Oropharyngeal candidiasis is one of the common manifestations seen in cancer patients on cytotoxic therapy and invasion into deeper tissues can occur if not treated promptly. Emergence of antifungal drug resistance is of serious concern owing to the associated morbidity and mortality. The present study aims at evaluation of clinicomycological association and antifungal drug susceptibility among the 180 recruited patients with cancer on chemotherapy and/or radiotherapy with signs or symptoms suggestive of oral candidiasis. Speciation and antifungal susceptibility was done by Microbroth dilution method for fluconazole, Itraconazole, and Amphotericin B as per standard microbiological techniques. Chi-square test was used for statistical analysis (*p* < 0.05 was considered statistically significant).* Candida albicans* was the predominant species isolated (94) (58%) followed by* Candida tropicalis* (34) (20.9%). Fluconazole and Itraconazole showed an overall resistance rate of 14% and 14.8%, respectively. All the isolates were susceptible to Amphotericin B. There was a significant association between the presence of dry mouth and isolation of* Candida *(*p* < 0.001). Such clinicomicrobiological associations can help in associating certain symptoms with the isolation of* Candida*. Species level identification with in vitro antifungal susceptibility pattern is essential to choose the appropriate drug and to predict the outcome of therapy.

## 1. Introduction

Oropharyngeal candidiasis is a common fungal infection in immunocompromised individuals. Conditions like malignancies, chemotherapy, and radiotherapy compromise the cell mediated immunity predisposing the person to fungal infections [1].* Candida* species are normally present as commensals in the oral cavity and their transition to become an opportunistic infective agent may be associated with certain virulence determinants [2]. Incidence of oral candidiasis has been reported to be ranging from 7 to 52% among cancer patients (head and neck malignancy, hematopoietic malignancy, and solid tumors) on chemotherapy and or radiotherapy [1].

A higher incidence of oral colonisation with non-*Candida albicans* has been reported in patients with advanced stage of cancer [3]. Although* Candida albicans* and non-*Candida albicans* are closely related, they differ in the antifungal susceptibility patterns. The colonised* Candida* can invade the underlying mucosa and enter the blood stream leading onto disseminated disease with considerable morbidity and mortality if not treated promptly. Fluconazole is one of the first line drugs used for the treatment of oral candidiasis in cancer patients [4, 5]. Amphotericin B is usually used for invasive* Candida* infections. Newer drugs like echinocandins are reserved for therapy of refractory candidiasis [4, 5]. It is of vital importance that cancer patients should be evaluated clinically and microbiologically for the presence of* Candida* in the oral cavity. The present study aims at speciation of the* Candida* isolated from oral cavity of patients with malignancy, to study the antifungal susceptibility pattern of the isolates and to evaluate the association between clinical and mycological findings.

## 2. Materials and Methods

This observational study was conducted in the Department of Microbiology, Government Kilpauk Medical College, Chennai, India, during the period of one year (Jan 2013 to Jan 2014). The study was approved by the institutional ethical committee. Cancer patients on chemotherapy and/or radiotherapy attending either outpatient or inpatient oncology clinic with signs and symptoms suggestive of oral candidiasis like presence of white plaque, erythematous lesion, ulcerative lesion, dryness of mouth, pain, altered taste sensation, and halitosis were included in the study. Unwillingness to participate and patients on antifungal therapy for past two weeks were excluded from the study. The study was explained and informed consent was obtained from the patients. The demographic data, present and past clinical history (type of cancer and treatment details), and complaints like presence of white patch in the oral cavity, pain, and erythematous lesion were documented in a pro forma for each patient. The data from the pro forma were analysed and tabulated and the association between the clinical information and the mycological findings was assessed and evaluated.

Two sterile swabs were used to collect sample from the oral cavity by swabbing over the lesions. The lesions (white patch, erythematous, and ulcerative lesion) were present over the buccal mucosa, tongue, and gingival regions and over the palatal regions in some cases. One swab was used for direct gram staining to look for the presence of gram positive yeast cell and pseudohyphae. The other swab was used for inoculating the specimen into Sabouraud dextrose agar and incubated at 24°C for 48 hours. The growth of creamy white colonies was subjected to gram staining for presence of gram positive budding yeast cells. Colonisation is defined as the presence of yeast cells in the oral cavity with/without clinical signs and symptoms. Infection or oral candidiasis is defined as the demonstration of gram positive hyphae/pseudohyphae and yeast cells microbiologically along with clinical signs and symptoms. Germ tube test was performed for all the isolates and further speciation was done by colony morphology in chrom agar (color of the colony), growth in corn meal agar (dalmau plate culture), and sugar assimilation and fermentation test as per standard microbiological techniques [6].

### 2.1. Antifungal Susceptibility Testing

Antifungal susceptibility testing was performed by Microbroth dilution method using RPMI (Roswell Park Memorial Institute) Medium 1640 with glutamine as per CLSI guidelines (2009) [7, 8]. Stock suspension was prepared and diluted with RPMI Medium to obtain a final inoculum size of 1 × 10^3^ to 5 × 10^3^/CFU/mL. The stock solutions were prepared by dissolving fluconazole powder in sterile distilled water. Amphotericin B and Itraconazole were dissolved in dimethyl sulfoxide. The drug (2x concentration) was dispensed into the wells of the sterile disposable Microtitre plate (100 *μ*L volume from row 1 to 10) with the highest drug concentration in row 1 and lowest concentration in row 10. Each well was inoculated with the 100 *μ*L of 2x inoculum suspension. The growth control well contains sterile drug-free medium and the corresponding inoculum suspension. The drug-free medium was added to row 11 to act as a growth control. The Microtitre plates were incubated at 35°C for 48 hours. The plates were observed for the presence or absence of visible growth. A numerical score which ranges from 0 to 4 was given to each well [7, 8]:

 0: optically clear.
 1: slightly hazy or approximately 25% of growth control.
 2: prominent decrease in turbidity or approximately 50% of growth control.
 3: slight reduction in turbidity or approximately 80% of growth control.
 4: no reduction in turbidity.

*End Point of MIC.* End point of MIC was considered as follows:

 Fluconazole and Itraconazole; score 2 or less,
 Amphotericin B: score 0.

The results are interpreted as per CLSI guidelines 2009.

### 2.2. Statistical Analysis

The sensitivity, specificity, positive predictive value, and negative predictive value of direct gram staining versus the culture positivity and germ tube test positivity in identifying* Candida albicans* were calculated. The significance of association between the symptoms/signs and the isolation of* Candida* was analysed by chi-square test and a two-sided *p* value less than 0.05 was considered statistically significant (Graphpad Quick Calcs software).

## 3. Results

**Figure 1:**
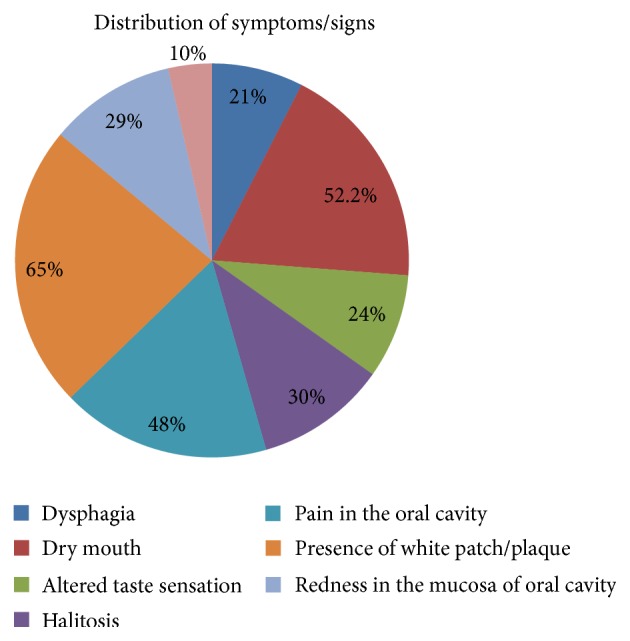
Distribution of symptoms and signs among the cancer patients.

A total of 192 cancer patients were initially recruited out of which twelve were excluded (eight patients were unwilling to participate and the rest were on antifungal therapy). Out of the 180 patients included in the study, male patients comprised 58.3% and females encompassed 41 (Table 1). The details regarding the distribution of cases by risk factors are depicted in Table 1. Patients with oral cancer comprised the major percentage of cases followed by gastrointestinal tract (GIT) malignancy (Table 1). All the cancer patients were on either chemotherapy or radiotherapy. The clinical symptoms and signs were analysed. The most commonly encountered symptom in the present study was dryness of mouth followed by pain in the oral cavity (Figure 1). The most common sign observed was presence of white plaques/patches and redness in the mucosa of oral cavity (Figure 1). The association between clinical details such as signs, symptoms, and mycological findings was evaluated and it was found that there was a significant association between dryness of mouth and isolation of* Candida* from the oral cavity (*p* < 0.001) (Table 2).

**Figure 2:**
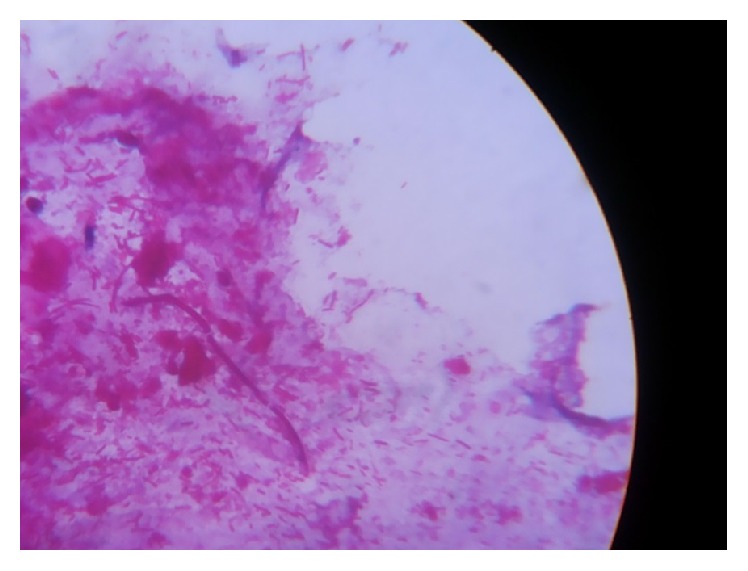
Direct gram staining showing the presence of gram positive hyphae.

In the present study, 90% of the cases showed direct gram staining positivity (presence of gram positive yeast cells and pseudohyphae) (Figure 2). The specimens that are culture positive were further speciated by germ tube test (for differentiating* Candida albicans* from non-*Candida albicans*), growth in chrom agar, corn meal agar, sugar assimilation, and fermentation tests.

The sensitivity, specificity, negative predictive value, positive predictive value, and diagnostic accuracy of gram staining versus culture positivity and positivity of germ tube test to identify* Candida albicans* are depicted in Table 3. Gram staining showed a sensitivity of 90%. Germ tube test showed sensitivity and specificity of 95%. Out of the 180 specimens received 152 (88.3%) were culture positive for* Candida*. Oral* Candida* infection was seen highest among patients with carcinoma of oral cavity (68) (89%) followed by carcinoma of gastrointestinal tract (34) (68%).* Candida albicans* (94) (58%) was the predominant species isolated followed by* Candida tropicalis* (34) (20.9%),* Candida glabrata* (14) (8.6%),* Candida krusei* (10) (6.17%),* Candida parapsilosis* (6) (3.7%), and* Candida kefyr* (4) (2.46%). Mixed infection (isolation of two species of* Candida*) was seen in 10 patients (Table 4).

Antibiotic susceptibility pattern was performed by Microbroth dilution method (Table 5). The overall resistance percentage for fluconazole and Itraconazole was 14% and 14.8%, respectively. Fluconazole showed a resistance of 5.8% and 12% for* Candida albicans* and* Candida tropicalis*, respectively. Itraconazole showed a resistance of 12% for fluconazole and Itraconazole each for* Candida albicans* and* Candida tropicalis*. None of the isolates of* Candida krusei* was susceptible to fluconazole.* Candida glabrata* showed the highest resistance for fluconazole (21.5%) and Itraconazole (14.3%). All the isolates were susceptible to Amphotericin B. The present study has documented that non-*Candida albicans* showed a higher percentage of resistance compared with* Candida albicans*.

## 4. Discussion

Oral candidiasis is a major problem in the world especially among cancer patients on cytotoxic therapy. The prevalence of oropharyngeal candidiasis was reported to be 38% by Ramirez-Amador et al. among cancer patients on radiotherapy [9]. Studies have reported the incidence of oral candidiasis to be ranging from 7 to 52% in cancer patients on chemotherapy and/or radiotherapy [1]. Patients usually progress from asymptomatic colonisation stage to infection. Conditions like malignancy, chemotherapy, and radiotherapy compromise the immunity and make the patient vulnerable to oropharyngeal candidiasis. The various other risk factors are use of antibacterials and steroids, comorbid illness like diabetes, poor oral hygiene, and tobacco usage [10].

*Candida* infection in patients with malignant diseases can lead to invasive infection and candidemia. The change in the etiology of oral candidiasis from* Candida albicans*, the commonly encountered species, to non-*Candida albicans* like* Candida glabrata* and* Candida krusei*, the more inherently drug resistant species, is particularly challenging for choosing the antifungal drug. Fluconazole is the first line of drug used to treat fungal infections in head and neck cancer [11]. Increase in resistance to fluconazole is being reported among cancer patients [1, 5, 12]. In the present study we have evaluated the association of clinicomicrobiological findings among cancer patients with oral candidiasis and the antifungal susceptibility pattern of the isolates.

The common cancer type encountered was carcinoma of oral cavity followed by malignancy of gastrointestinal tract (GIT) similar to what is reported by Afraseyabi et al. [3]. Dryness of mouth and pain in the oral cavity are the most frequently encountered symptoms. In the present study we found out that there was a significant association between the presence of dry mouth and isolation of* Candida* species (*p* < 0.001). Alt-Epping et al. have reported a similar association between dryness of mouth and isolation of* Candida* [13]. Dryness of mouth can occur as a result of chemotherapy or radiotherapy and can cause mucosal disruption facilitating infection by* Candida* [13]. The association between the presence of a symptom and the isolation of* Candida* is not causal all the time. However, from a clinical viewpoint, associating certain clinical signs and symptoms with the microbiological findings will be helpful to ascertain the affliction of the sign/symptom and a necessity to identify and treat the cause. Such associations might be useful clinically.

Studies by Nadagir et al. and Lattif et al. have showed a direct gram staining positivity rate of 75% compared to 90% in the present study [14, 15]. Direct gram staining of the specimen along with the clinical signs and symptoms for oral candidiasis can be a valuable tool in differentiating colonisation from infection. In the present study, 95% of the* Candida albicans* showed germ tube test positivity. Enwuru et al. and Srinivasan and Kenneth have reported a germ tube positivity of 96.7% and 89% among* Candida albicans*, respectively [16, 17]. Although germ tube test, a simple rapid test, offers 95% consistency for identifying* Candida albicans*, it must be used in concurrence with other phenotypic tests for species identification. Oral candidial infection was seen highest among patients with carcinoma of oral cavity (68) (89%), similar to Lone et al., followed by carcinoma of gastrointestinal tract 34 (68%).

*Candida albicans* was the predominant species isolated followed by* Candida tropicalis*, similar to Schelenz et al. and Safdar et al. (74% and 67.3% were* Candida albicans*, resp.) [18, 19]. Studies have reported* Candida glabrata* as the commonly isolated non-*Candida albicans* among cancer patients [5, 19]. Oral colonisation with non-*Candida albicans* occurs in higher rates in cancer patients [2].

With the usage of azoles for the empirical treatment of candidial infection, there has been a rise in the incidence of non-*Candida albicans* like* Candida krusei* and* Candida glabrata* with reduced susceptibility to azole antifungal agents [4]. Hence, it is essential to regularly investigate the antifungal resistance pattern to get up-to-date information which will help the physician in selecting the antifungal drug for empirical therapy. The overall resistance for fluconazole and Itraconazole in the present study was 14.1% and 14.8%, respectively. Literature from across the world has reported fluconazole and Itraconazole resistance to be ranging from 2% to 10% and from 9% to 10%, respectively [5, 18, 19]. The resistance rate for fluconazole and Itraconazole in the present study was high compared to the above-cited studies.

In the present study, for* Candida albicans* the resistance for fluconazole and Itraconazole was 6% and 12.3%, respectively, similar to Schelenz et al. and Safdar et al. [18, 19]. In the present study, for* Candida tropicalis*, resistance for fluconazole and Itraconazole was 12%. Safdar et al. has reported fluconazole and Itraconazole resistance as 19% and 21%, respectively, for* Candida tropicalis* among cancer patients [19].

*Candida glabrata* showed a resistance of 21.5% and 14.3% for fluconazole and Itraconazole, respectively. In contrast to our study, Safdar et al. have reported a high resistance among* Candida glabrata* for fluconazole and Itraconazole (30.8% and 46.2%, resp.). Bagg et al. have reported a resistance as high as 78.7% for fluconazole among* Candida glabrata* [2]. For all patients with culture positivity, the antifungal susceptibility pattern was informed to the treating physician and oral fluconazole therapy was given for 112 patients along with Clotrimazole/Amphotericin B oral lozenges. In case of fluconazole resistance, Itraconazole/Clotrimazole were prescribed. Follow-up was lost for the rest of forty patients. The limitation of the present study was that the previous oral* Candida* colonisation status of the recruited patients prior to the initiation of chemotherapy and/or radiotherapy was not known and whether the colonised* Candida* species were implicated in causing the present infection was also not assessed.

In the present study, non-*Candida albicans* have demonstrated a higher percentage of resistance compared to* Candida albicans* for the empirically used drugs like fluconazole and Itraconazole. High incidence of oral colonisation and infection with such inherently drug resistant isolates becomes more challenging for choosing the prophylactic drug. Such drug resistant isolates can invade underlying mucosa and enter the blood stream causing invasive infections. Prevalence of fungal infections has increased several times among individuals with lowered immune status such as cancer patients on chemotherapy and radiotherapy [4]. Cytotoxic therapy causes dryness of oral mucosa facilitating infections by various pathogens. Studies have reported that development of candidiasis is a two-step process consisting of colonisation and subsequent invasion of epithelial layer [4, 20]. Once colonisation has been established, impaired cellular immunity permits invasion of epithelial layer. Neutropenia, irradiation, and chemotherapy will lead to mucosal disruption facilitating deeper invasion by* Candida* [4].

The emergence of antifungal resistance within* Candida* species particularly in cancer patients is of serious concern because such drug resistant isolates may invade the deeper tissues leading to disseminated infection. The high prevalence of* Candida* in the oral cavity of cancer patients treated by chemotherapy/radiotherapy necessitates the need for routine periodic surveillance of fungal infections to determine the antifungal resistance pattern.

## 5. Conclusion

Oral candidiasis is a common fungal infection in patients with cancer on treatment with chemotherapy and/or radiotherapy. The association between clinical and microbiological findings can help in the correlation of certain symptoms with the isolation of* Candida* among cancer patients in certain instances, but such associations are not always causal. From a clinical perspective it might be useful if the patient complains of that particular symptom like dry mouth; alertness for associated* Candida* infection should be high.* Candida albicans* and non-*Candida albicans* differ significantly in their antifungal susceptibility pattern. Non-*Candida albicans* like* Candida krusei* are inherently resistant to azoles. Hence, species level identification with the in vitro antifungal susceptibility pattern is essential to choose the appropriate antifungal drug and to predict the outcome of therapy.

## Figures and Tables

**Table 1:** Demographic data and the distribution of patients based on cancer type.

	Number
Age	
0–20	20 (11.1%)
21–40	44 (24.4%)
41–60	64 (35.5%)
61–70	52 (28.8%)

Sex-wise distribution	
Male	105 (58.3%)
Female	75 (41.6%)

Distribution of the patients based on the type of cancer	
Carcinoma oral cavity	76 (42.2%)
Carcinoma stomach	26 (14.4%)
Carcinoma esophagus	22 (12.2%)
Lymphoma	16 (8.8%)
Carcinoma cervix	14 (7.7%)
Chronic myeloid leukemia	12 (6.6%)
Bone tumor	8 (4.4%)
Carcinoma bladder	4 (2.2%)
Carcinoma colon	2 (1.1%)

**Table 2:** Mycological and clinical association between the symptoms/signs.

S. number	Symptom/signs	No isolation of candida	Isolation of candida	Total	*p* value
1	Presence of dry mouth	68	26	94	<0.001
	Absence of dry mouth	84	2	86	

2	Presence of erythema	41	11	52	0.187
	Absence of erythema	111	17	128	

3	Presence of white patch	20	119	139	0.25
	Absence of white patch	7	44	51	

4	Presence of ulcer	6	12	18	—
	Absence of ulcer	146	16	162	

**Table 3:** Sensitivity, specificity, negative predictive value, positive predictive value, and the diagnostic accuracy of (a) direct gram staining versus culture positivity and (b) germ tube test positivity in identifying *Candida albicans*.

Parameter	Sensitivity	Specificity	Negative predictive value	Positive predictive value	Diagnostic accuracy
Direct gram staining	90.06%	100%	52.63%	100%	91.04%
Germ tube test	95.68%	95.40%	87.50%	98.52%	95.63%

**Table 4:** Species-wise distribution of the isolates.

S. number	Species	Percentage
1	*Candida albicans*	94 (58%)
2	*Candida tropicalis*	34 (20.9%)
3	*Candida glabrata*	14 (8.6%)
4	*Candida krusei*	10 (6.17%)
5	*Candida parapsilosis*	6 (3.7%)
6	*Candida kefyr*	4 (2.46%)

	Total	162

Mixed infection was seen in 10 patients (isolation of two *Candida* species).

**Table 5:** Antifungal susceptibility pattern by Microbroth dilution method.

S. number	Antifungal drug	Fluconazole resistant	Fluconazole SDD^*∗*^	Itraconazole resistant	Itraconazole SDD	Amphotericin B susceptible
	MIC^§^ range	>8 *µ*g/mL	16–32 *µ*g/mL	>0.125 *µ*g/mL	0.25–0.5 *µ*g/mL	<1 *µ*g/mL

1	*Candida albicans* *n* = 94	6 (5.8%)	23 (24.4%)	12 (12.7%)	20 (21.27%)	94 (100%)

2	*Candida tropicalis* *n* = 34	4 (11.7%)	10 (29.4%)	4 (12%)	11 (32.3%)	34 (100%)

3	*Candida glabrata* *n* = 14	3 (21.4%)	4 (28.5%)	4 (28.5%)	5 (35.7%)	14 (100%)

4	*Candida krusei* *n* = 10	10 (100%)	0	4 (60%)	2 (20%)	10 (100%)

5	*Candida parapsilosis* *n* = 6	0	0	0	0	6 (100%)

6	*Candida kefyr* *n* = 4	0	0	0	0	4 (100%)

	Overall resistance%	23 (14.1%)	37 (22.8%)	22 (14.8%)	38 (23.4%)	Nil

SDD^*∗*^: susceptible dose dependent.

MIC^§^: minimum inhibitory concentration.

## References

[1] Lone M. S., Bashir G., Bali N. (2014). Oral Candida colonization and infection in cancer patients and their antifungal susceptibility in a tertiary care hospital. *International Journal of Advanced Research*.

[2] Bagg J., Sweeney M. P., Lewis M. A. O. (2003). High prevalence of non-albicans yeasts and detection of anti-fungal resistance in the oral flora of patients with advanced cancer. *Palliative Medicine*.

[3] Afraseyabi Sh., Afkhamzadeh A., Sabori H., Verdi F., Khaksar N., Mosavei B. (2011). Oral candidiasis amongst cancer patients at Qods hospitals in Sanandaj. *African Journal of Clinical and Experimental Microbiology*.

[4] Lalla R. V., Latortue M. C., Hong C. H. (2010). A systematic review of oral fungal infections in patients receiving cancer therapy. *Supportive Care in Cancer*.

[5] Shokohi T., Bandalizadeh Z., Hedayati M. T., Mayahi S. (2011). In vitro antifungal susceptibility of *Candida* species isolated from oropharyngeal lesions of patients with cancer to some antifungal agents. *Jundishapur Journal of Microbiology*.

[6] Adhikary R., Joshi S. (2011). Species distribution and anti-fungal susceptibility of Candidaemia at a multi super-specialty center in Southern India. *Indian Journal of Medical Microbiology*.

[7] Diekema D. J., Messer S. A., Boyken L. B. (2009). In vitro activity of seven systemically active antifungal agents against a large global collection of rare *Candida* species as determined by CLSI broth microdilution methods. *Journal of Clinical Microbiology*.

[8] Clinical and Laboratory Standards Institute (CLSI) (2009). Reference method for broth dilution antifungal susceptibility testing of yeasts. Approved standard, 3rd ed.. *CLSI Document*.

[9] Ramirez-Amador V., Silverman S., Mayer P., Tyler M., Quivey J. (1997). Candidal colonization and oral candidiasis in patients undergoing oral and pharyngeal radiation therapy. *Oral Surgery, Oral Medicine, Oral Pathology, Oral Radiology, and Endodontics*.

[10] Bensadoun R.-J., Patton L. L., Lalla R. V., Epstein J. B. (2011). Oropharyngeal candidiasis in head and neck cancer patients treated with radiation: update 2011. *Supportive Care in Cancer*.

[11] Redding S. W., Zellars R. C., Kirkpatrick W. R. (1999). Epidemiology of oropharyngeal *Candida* colonization and infection in patients receiving radiation for head and neck cancer. *Journal of Clinical Microbiology*.

[12] Mañas A., Cerezo L., De La Torre A. (2012). Epidemiology and prevalence of oropharyngeal candidiasis in Spanish patients with head and neck tumors undergoing radiotherapy treatment alone or in combination with chemotherapy. *Clinical and Translational Oncology*.

[13] Alt-Epping B., Nejad R. K., Jung K., Groß U., Nauck F. (2012). Symptoms of the oral cavity and their association with local microbiological and clinical findings-a prospective survey in palliative care. *Supportive Care in Cancer*.

[14] Nadagir S. D., Chunchanur S. K., Halesh L. H., Yasmeen K., Chandrasekhar M. R., Patil B. S. (2008). Significance of isolation and drug susceptibility testing of non-Candida albicans species causing oropharyngeal Candidiasis in HIV patients. *Southeast Asian Journal of Tropical Medicine and Public Health*.

[15] Lattif A. A., Banerjee U., Prasad R. (2004). Susceptibility pattern and molecular type of species-specific candida in oropharyngeal lesions of indian human immunodeficiency virus-positive patients. *Journal of Clinical Microbiology*.

[16] Enwuru C. A., Ogunledun A., Idika N. (2008). Fluconazole resistant opportunistic oro-pharyngeal candida and non-candida yeast-like isolates from HIV infected patients attending ARV clinics in Lagos, Nigeria. *African Health Sciences*.

[17] Srinivasan L., Kenneth J. (2006). Antibiotic susceptibility of *Candida* isolates in a tertiary care hospital in Southern India. *Indian Journal of Medical Microbiology*.

[18] Schelenz S., Abdallah S., Gray G. (2011). Epidemiology of oral yeast colonization and infection in patients with hematological malignancies, head neck and solid tumors. *Journal of Oral Pathology and Medicine*.

[19] Safdar A., Chaturvedi V., Cross E. W. (2001). Prospective study of *Candida* species in patients at a comprehensive cancer center. *Antimicrobial Agents and Chemotherapy*.

[20] Delsing C. E., Bleeker-Rovers C. P., van de Veerdonk F. L. (2012). Association of esophageal candidiasis and squamous cell carcinoma. *Medical Mycology Case Reports*.

